# Supplementary material for: The mechanism of a high-affinity allosteric inhibitor of the serotonin transporter
Source: Nat Commun. 2020 Mar 20;11:1491. doi: 10.1038/s41467-020-15292-y (PMC7083837; doi:10.1038/s41467-020-15292-y)
Supplement: Supplementary file 1 — Supplementary Information [file 41467_2020_15292_MOESM1_ESM.pdf]

Supplementary Information

Plenge et al.

The mechanism of a high-affinity allosteric inhibitor to the serotonin transporter

---

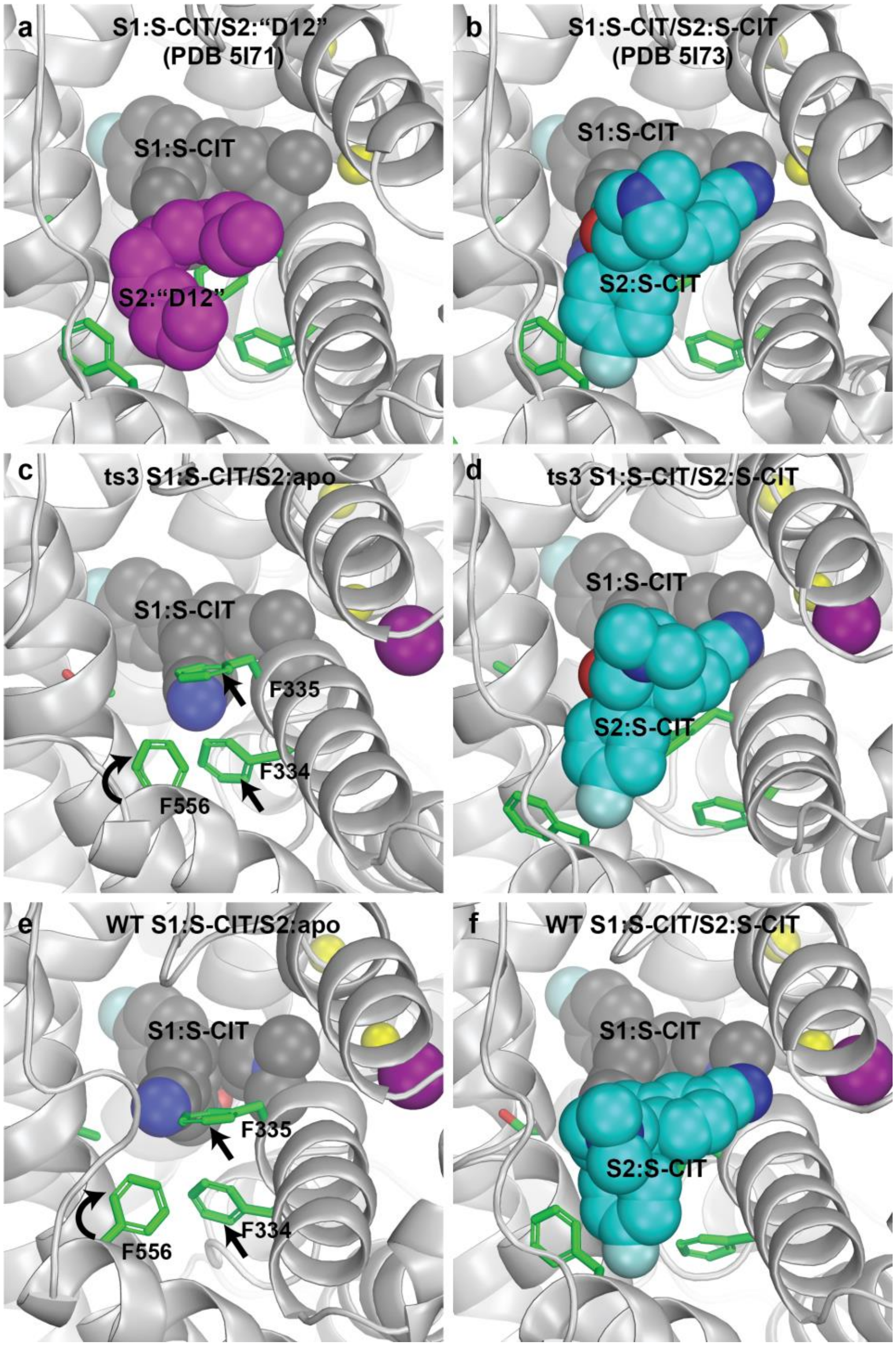

Supplementary Figure 1. Phe335, Phe334, and Phe556 rearrange in the absence of S-CIT or the “dodecane” molecule in the S2 site, Panels **a** and **b** show that in the 5I71 and 5I73 structures, the S2 site is occupied by a “dodecane” (D12, likely part of a lipid molecule used in the crystallization process) and S-CIT, respectively. In both ts3 (**c** and **d**) and WT (**e** and **f**) simulations, in the absence of these molecules (**c** and **e**), Phe335 moves towards center of the EV, while Phe556 rotates to face inward. These two residues form an aromatic cluster with Phe334.

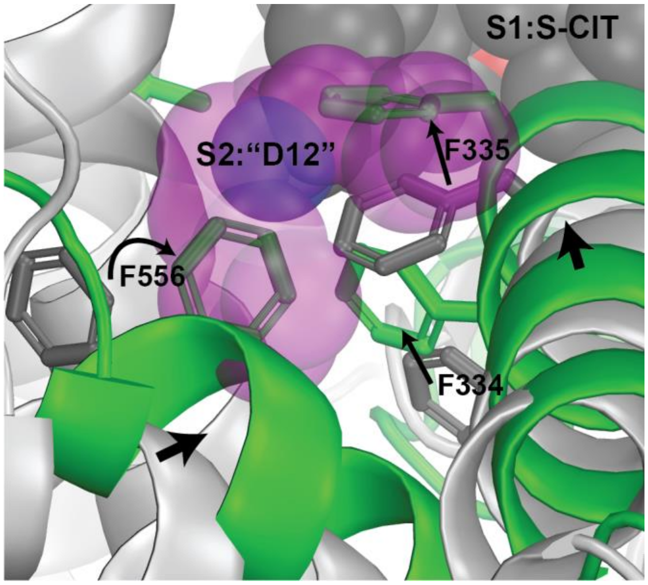

Supplementary Figure 2. Phe335 and Phe556 fill the space occupied by the "dodecane" in the S2 site. A representative frame of the ts3 S1:S-CIT/S2:*apo* simulations (green) is superimposed on the 5I71 structure (gray). The rearranged residues Phe335 and Phe556 fill the space in the S2 site occupied by "dodecane" (purple) in the 5I71 structure.

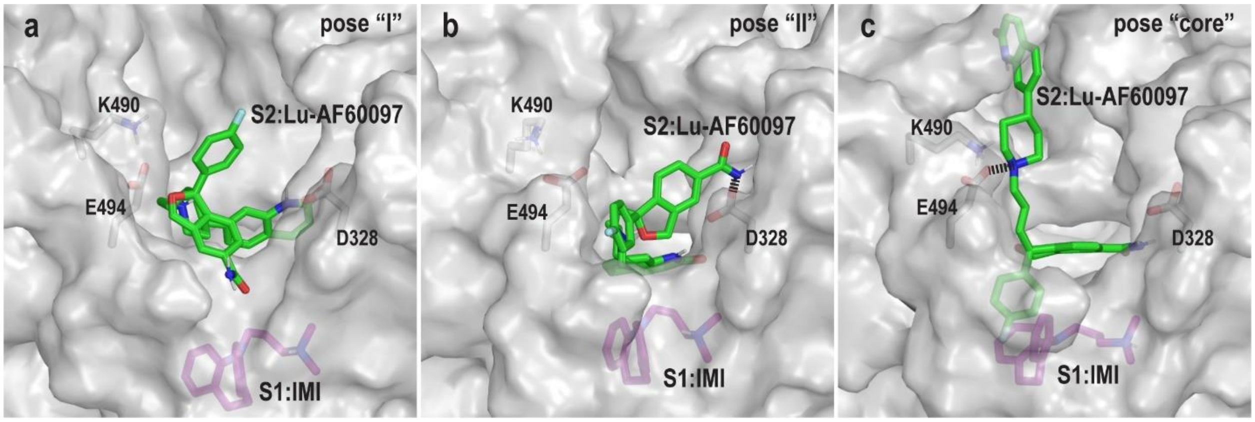

Supplementary Figure 3. Comparison of three simulated S2:Lu AF60097 poses. (**a**) Pose “I” is not fully engaged with the extracellular vestibule (EV) of hSERT. (**b**) Pose “II” forms a polar interaction with Asp328, however, the mutation of this residue does not significantly affect Lu AF60097 potency (Table 2). (c) Pose “core” is fully accommodated by EV and forms favored interactions with Lys490 and Glu494, consistent with the mutagenesis results (see text and Table 2). Lu AF60097 is shown in green sticks, whereas hSERT is shown in a transparent gray surface representation.

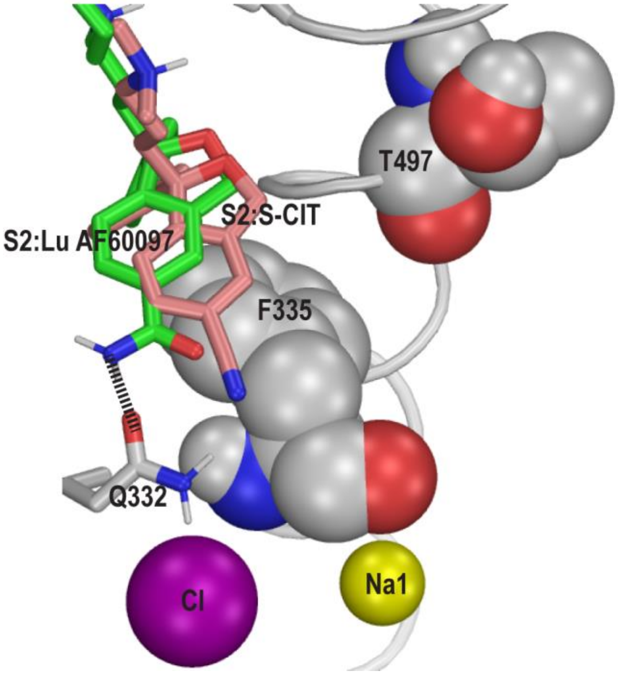

Supplementary Figure 4. Binding of S2 ligands correlates with the conformations of Thr497 and Phe335. In the presence of S1:IMI, superposition of the S2:Lu AF60097 S2:S-CIT binding poses shows the rearrangement of the benzofuran ring in S2:AF60097 compared to that S2:S-CIT. Thr497 and Phe335 are shown in spheres, S2:AF60097, S2:S-CIT, and Gln332 are green, salmon, and gray in sticks, respectively, and Na1 and Cl ions are in yellow and purple spheres, respectively.

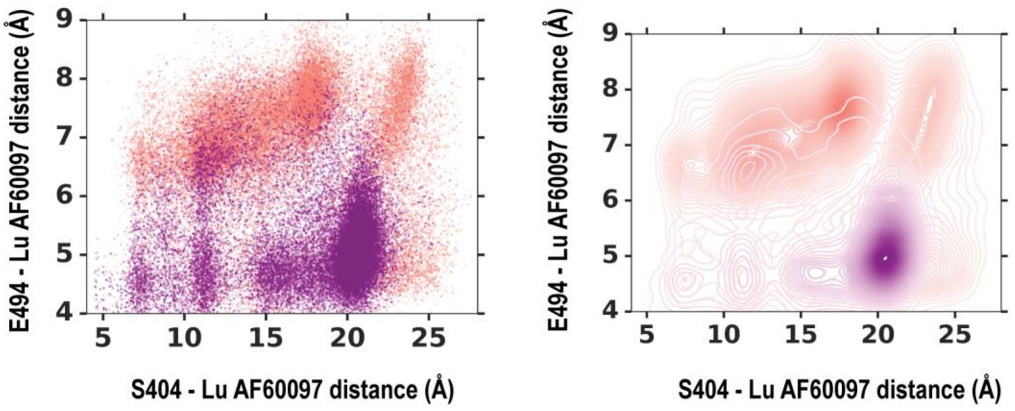

Supplementary Figure 5. When the quinolinone moiety of S2:Lu AF60097 does not protrude into the sub-pocket near EL4b, it occupies distinct conformational space in the EV of hSERT depending whether S-CIT or IMI is in the S1 site. The distances of Lu AF60097 2-oxo modification and the side chain of Ser404 are plotted against the distances of Lu AF60097 charged N and Glu494 side chain for the S1:IMI/S2:Lu AF60097 (purple) and S1:S-CIT/S2:Lu AF60097 (salmon) conditions with the distribution (left panel) and contour (right panel) plots. Thus, it is unlikely that the inability of the quinolinone moiety of S2:Lu AF60097 to protrude into that sub-pocket is due to inadequately sampling of the MD simulations.

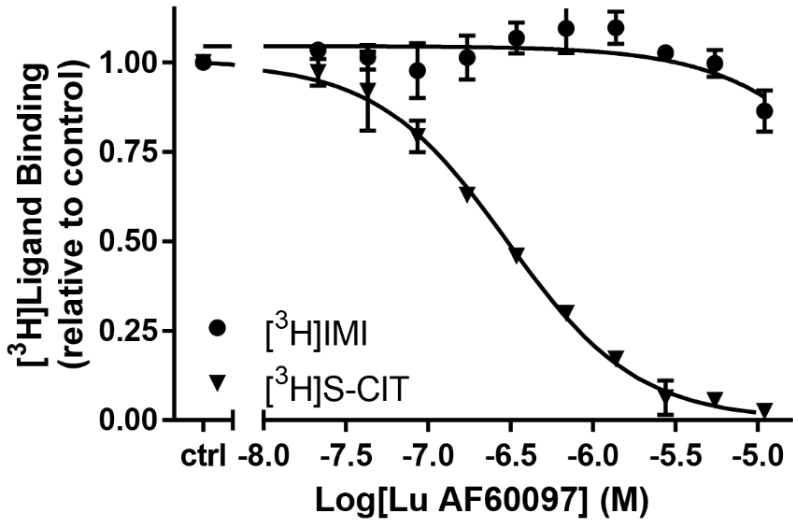

Supplementary Figure 6. Equilibrium binding experiment for Lu AF60097 displacement of either [^{3}H]IMI (circles) or [^{3}H]S-CIT (triangles). The experiment is performed on membrane preparations from COS-7 cells transfected with SERT WT. Data are shown as mean±SE (error bars) of 3 experiments performed in triplicates. Source data are provided as a Source Data file.

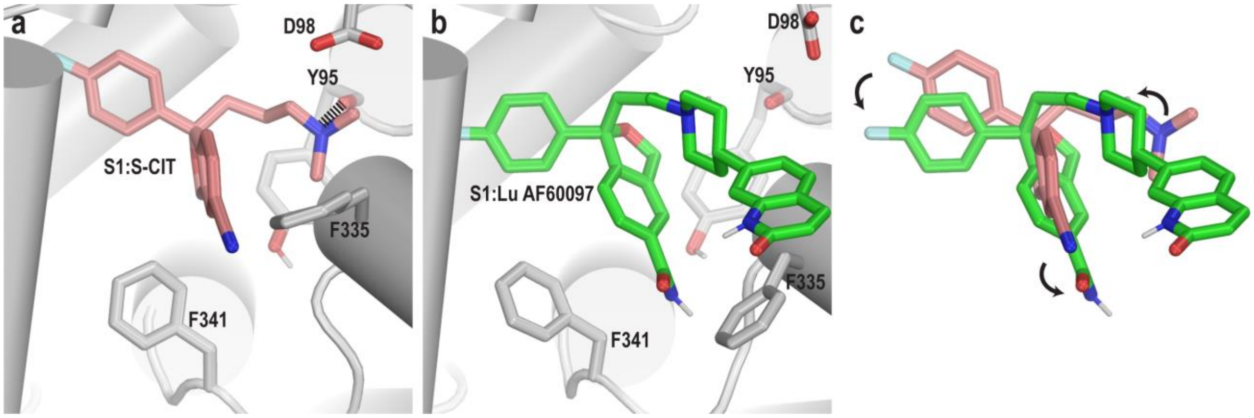

Supplementary Figure 7. S1:Lu AF60097 has a tilted pose compared to that of S1:S-CIT and does not form any ionic interaction with SERT. In the WT SERT models, the formation of an ionic interaction between the tertiary amine of S1:S-CIT and Tyr95 in the S1:S-CIT/S2:*apo* condition (**a**) is indicated by a dotted line, S1:Lu AF60097 does not form any ionic interaction with SERT in the S1:Lu AF60097/S2:*apo* condition (**b**). The poses of S1:S-CIT and S1:Lu AF60097 are superimposed in panel **c** to demonstrate the tilting of the S-CIT scaffold of S1:Lu AF60097 compared to S1:S-CIT. Ligands are colored in the same scheme as Figs. 2 and 3.

Supplementary Table 1. Summary of molecular dynamics simulations

construct	S1 ligand	S2 ligand (pose)	number of trajectories	lengths (µs)
ts3	S-CIT	*apo*	4	7.80
		S-CIT	8	10.20
WT	S-CIT	*apo*	11	18.03
		S-CIT	6	13.62
		Lu AF60097 (core)	10	18.69
	IMI	*apo*	8	12.24
		S-CIT	6	9.24
		Lu AF60097 (I)	2	2.40
		Lu AF60097 (II)	2	2.40
		Lu AF60097 (core)	15	28.26
	Lu AF60097	*apo*	2	2.40
**Total**			**74**	**125.28**

Supplementary Table 2. Allosteric potency for S- and R-CIT to SERT E494Q and T497A

Construct	Compound	Allosteric Potency<br>Inhibition of [^{3}H]S-CIT dissociation<br>(nM)	n	Allosteric Potency<br>Inhibition of [^{3}H]IMI dissociation<br>(nM)	n
SERT E494Q	S-citalopram	64800 [56600; 74100]	5	131000 [123000; 140000]	6
	R-citalopram	137000 [118000; 158000]	5	199000 [191000; 207000]	5
SERT T497A	S-citalopram	5100 [3200; 8000]	3	8800 [5900; 13400]	3
	R-citalopram	17800 [15700; 20200]	3	11600 [10100; 13300]	3

Allosteric potencies are the IC_{50} from non-linear regression analysis of the change in dissociation rate constant relative to no compound present ($k_{[cmpd]}/k_{buffer}$) as a function of the added compound concentration (Log[cmpd]), here either S-CIT or R-CIT. Experiments performed on membrane preparations of COS-7 cells expressing the indicated SERT mutants. Data are shown as mean [SE interval] and are calculated from pIC50 and the SE interval from pIC50±SE.

Supplementary Table 3. The Lu AF60097 interacting residues and their interacting frequencies in the S1:IMI/S2:Lu AF60097 condition. In an MD frame, if the shortest heavy-atom distance between S2:Lu AF60097 and any given SERT residue was within 5 Å, we defined that S2:Lu AF60097 forms an interaction with this residue. The interaction frequencies were calculated based on 5000 random frames in the S1:IMI/S2:AF60097 condition. The residues below are those having >50% frequencies.

**segment**	**residue**	**Interaction frequency (%)**
**TM1**	ARG104	100.0
	ILE108	95.0
**EL2**	TYR232	50.8
**TM6**	ASP328	67.6
	ALA331	100.0
	GLN332	100.0
	PHE335	100.0
**EL4**	ASP400	100.0
	ALA401	100.0
	GLY402	100.0
	PRO403	57.2
	SER404	99.9
**TM10**	LYS490	99.9
	GLU493	100.0
	GLU494	100.0
	GLY498	99.9
	PRO499	100.0
	LEU502	100.0
**TM11**	ILE552	99.6
	ILE553	80.6
	PHE556	100.0
**EL6**	LEU565	50.0
	PHE566	50.1

Supplementary Table 4. Effect of Lu AF60097 on 5-HT transport

[Lu AF60097] (nM)	V_{max} 5-HT (fmol/min)	K_M (nM)
0	1183 ± 23	475 [392; 576]
10	1232 ± 48	519 [434; 619]
20	1279 ± 64	525 [375; 736]
40	1007 ± 58	438 [358; 536]
80	1361 ± 78	1080 [870; 1350]
160	1235 ± 34	904 [862; 950]
320	973 ± 25	1000 [923; 1090]
640	971 ± 21	1660 [1510; 1830]

Data are calculated from non-linear regression analysis form saturation uptake experiments for [^{3}H]5-HT transport as a function of increasing Lu AF60097 concentrations (0 to 640 nM) as shown in Fig 5b. Experiments are performed on intact COS-7 cells transiently expressing SERT WT. V_{MAX} values are shown as mean ± SE and K_M values as mean [SE interval] calculated from pIC50±SE of at least three independent experiments.

Supplementary Table 5. Effect of Lu AF60097 and imipramine alone or in combination on [^{3}H]5-HT transport velocity

Condition	[^{3}H]5-HT uptake (in % of buffer)	n
Buffer	100	5
+IMI (4 nM)	89.3 ± 0.8	7
+ Lu AF60097 (27 nM)	95 ± 1.8	7
+IMI +Lu AF60097	64.7 ± 0.3	5

Inhibition of [^{3}H]5-HT uptake into intact COS-7 cells transiently expressing SERT WT by the indicated concentrations of inhibitors. All experiments are performed in triplicates on intact COS-7 cells transiently transfected with SERT WT. Data are shown as means ± SE.

Supplementary Methods

Synthesis of target compounds Lu AF60097, AF56461 and AF60098. Reagents and solvents were obtained from commercial sources and used as received or synthesized as described in the literature. Thin layer chromatography was carried out on Merck Silica gel 60 F254. Flash chromatography was performed using Merck Silica Gel 60 (40-63 µm). ^{1}H nuclear magnetic resonance (NMR) spectra were recorded at ambient temperature on a Bruker Avance AV-500 equipped with a 5 mm QNP probe with Z-gradients operating at 500.13 MHz or a Bruker 600-Avance-III spectrometer equipped with a 5 mm TCI cryoprobe with Z-gradient operating at 600.16 MHz. Chemical shifts (δ) are reported in parts per million relative to tetramethylsilane (TMS) or residual solvent, and coupling constants (J) are given in Hertz. The following abbreviations are used in reporting NMR data: s, singlet; brs, broad singlet; d, doublet; t, triplet; m, multiplet. High-resolution mass spectrometry (HRMS) was recorded on a Bruker Compact qTOF equipped with electrospray operating in positive mode. Internal calibration was performed on sodium formiate. The liquid chromatography (LC)-system was run on Waters Aquity UPLC with DAD, ELSD, and SQD MS detector with APPI source (method 450) or on Waters Aquity UPLC with DAD, ELSD, and TQD detector with APPI source (method 550). In all cases UV purity is reported at 254 nm. LC methods 450 and 550: Column Aquity APLC BEH B18, 2.1 x 50 mm, 1.7 µ particles. Flow rate 1.2 mL/min at 60 ^{o}C. Solvent A: Water + 0.05 % trifluoroacetic acid, Solvent B: Acetonitrile/water (95:5) + 0.03 % trifluoroacetic acid. Gradient: 0.00 min 90 % A; 1.0 min 0 % A; 1.2 min 90 % A; Total run time 1.2 min. Enantiomer ratio was determined on an Aurora Fusion A5 SFC system operating at 3 ml/min at 40 ^{o}C and 150 bar back pressure. UV-detection at 254 nm. Method A: column: LuxCellulose-1, 4.6 x 150 mm, 3 µ particles, 60 % CO_2 and 40 % methanol + 0.1% diethylamine. Method B: Column: Chiralcel-AD3, 4.6 x 150 mm, 3 µ particles, 60% CO_2 and 40% methanol + 0.1% diethylamine.

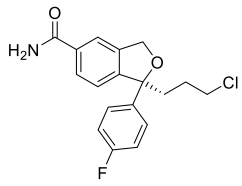

(*S*)-1-(3-Chloropropyl)-1-(4-fluorophenyl)-1,3-dihydroisobenzofuran-5-carboxamide. (*S*)-1-(3-chloropropyl)-1-(4-fluorophenyl)-1,3-dihydroisobenzofuran-5-carbonitrile[1] (1.00 g, 3.17 mmol) was dissolved in dimethyl sulfoxide (DMSO) (7.50 mL) and cooled to 16 °C in cold water. Potassium carbonate (3.06 g, 22.17 mmol) was added to the mixture and subsequently followed by a dropwise addition over 30 s of an aqueous 8.8 M hydrogen peroxide solution (1.60 mL, 14.25 mmol). Gas evolved from the reaction mixture which also turned more viscous. The mixture was stirred at 15-20 °C for 20 min and poured onto brine. The aqueous mixture was extracted with ethyl acetate, and the combined organic phase was washed with brine, dried using magnesium sulphate, filtered and concentrated under reduced pressure. The product that included approx. 20% ethyl acetate as estimated based on ^{1}H NMR was obtained as a colourless oil (0.95 g, 86%) and used in the next step without further purification. ^{1}H NMR (500 MHz, DMSO-d_6) δ 7.94 (s, 1H), 7.82 (d, 1H), 7.77 (s, 1H), 7.62-7.56 (m, 3H), 7.36 (s, 1H), 7.16 (t, 2H), 5.19 (d, 1H), 5.13 (d, 1H), 3.61 (t, 2H), 2.35-2.22 (m, 2H), 1.70-1.47 (m, 2H).

The following compounds were prepared in a similar manner and used in the next step without further purification.

(*R*)-1-(3-Chloropropyl)-1-(4-fluorophenyl)-1,3-dihydroisobenzofuran-5-carboxamide. Prepared starting from (*R*)-1-(3-chloropropyl)-1-(4-fluorophenyl)-1,3-dihydroisobenzofuran-5-carbonitrile (0.55 g, 1.74 mmol) that was prepared from *R*-citalopram in accordance with literature procedure[1] to yield title compound 0.64 g, 88%.

(*RS*)-1-(3-Chloropropyl)-1-(4-fluorophenyl)-1,3-dihydroisobenzofuran-5-carboxamide. Prepared starting from (*RS*)-1-(3-chloropropyl)-1-(4-fluorophenyl)-1,3-dihydroisobenzofuran-5-carbonitrile (2.00 g, 6.33 mmol) that was prepared from citalopram in accordance with literature procedure[1] to yield title compound 2.30 g, 98%.

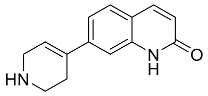

7-(1,2,3,6-Tetrahydropyridin-4-yl)quinolin-2(1H)-one.

7-bromoquinolin-2(1*H*)-one. 7-Bromo-2-chloroquinoline (25.0 g, 103 mmol) was dissolved in tetrahydropyridine (THF) (175 mL) and added slowly to a mixture of 12 M hydrochloric acid (250 mL) and water (250 mL). A thick suspension was formed, which was subsequently heated under reflux (approx. 80 °C) until a clear solution was formed. The clear solution was boiled under reflux for 3.5 h and gradually, a dense white precipitate formed. The mixture was added water (400 mL) and cooled to 5 °C on an ice bath. The precipitate was collected by filtration and dried under vacuo to yield 7-bromoquinolin-2(1*H*)-one as a cream-coloured powder (22.3 g, 97%) that was used in the next step without further purification.

tert-Butyl 7-Bromo-2-oxoquinoline-1(2*H*)-carboxylate. Sodium hydride (7.95 g, 199 mmol) was suspended in THF (450 mL) and 7-bromoquinolin-2(1*H*)-one (22.3 g, 99.0 mmol) was carefully added to the suspension over 15 min at 32-37 °C. The resulting mixture was stirred at 50 °C for 1 h and cooled on ice before adding a solution of di-*tert*-butyldicarbonate (43.4 g, 199 mmol) in THF (150 mL) over a period of 10 min at 7-8 °C. The resulting mixture was stirred at 40 °C for 30 min, and a thick suspension was gradually formed. The mixture was cooled on an ice bath and thereafter carefully poured on a saturated ammonium chloride solution. The aqueous mixture was extracted with ethyl acetate, and the combined organic phase was washed with brine, dried over magnesium sulphate, filtered, and concentrated in vacuo. The residual oil was purified by column flash chromatography (eluent: heptane/ethyl acetate 4:1) to afford a crude solid after concentration in vacuo. The solid was dissolved in ethyl acetate (100 mL) and heptane (125 mL) was added. The mixture was concentrated to a total of 75 mL and a precipitate started forming, which was collected by filtration and dried in vacuo to yield *tert*-butyl 7-bromo-2-oxoquinoline-1(2*H*)-carboxylate as a dense white powder (24.8 g, 77%) that was used in the next step without further purification.

tert-Butyl 7-(1-(*tert*-Butoxycarbonyl)-1,2,3,6-tetrahydropyridin-4-yl)-2-oxoquinoline-1(2*H*)-carboxylate. *tert*-Butyl 7-Bromo-2-oxoquinoline-1(2*H*)-carboxylate (12.0 g, 37.0

mmol) was dissolved in dimethyl formamide (DMF) (125 mL) and *tert*-butyl 4-(4,4,5,5-tetramethyl-1,3,2-dioxaborolan-2-yl)-1,2,3,6-tetrahydropyridine-1-carboxylate (14.2 g, 46.0 mmol) and potassium carbonate (7.67 g, 55.5 mmol) was added. To this mixture was added 1,1'-bis(diphenylphosphino)ferrocene-palladium(II) dichloride dichloromethane complex (3.02 g, 3.70 mmol) and the resulting mixture was heated at 60 °C in an oil-bath for 6 h and then stirred overnight at room temperature. The mixture was poured onto brine, and the aqueous phase was extracted with ethyl acetate. The combined organic phase was washed with brine, dried over magnesium sulphate, filtered and concentrated in vacuo. The residual oil was purified by column flash chromatography (eluent: heptane/ethyl acetate 2:1) to afford *tert*-butyl 7-(1-(*tert*-butoxycarbonyl)-1,2,3,6-tetrahydropyridin-4-yl)-2-oxoquinoline-1(2*H*)-carboxylate after concentration in vacuo (12.0 g, 76%) and used in the next step without further purification.

<u>7-(1,2,3,6-Tetrahydropyridin-4-yl)quinolin-2(1*H*)-one.</u> *tert*-Butyl 7-(1-(*tert*-Butoxycarbonyl)-1,2,3,6-tetrahydropyridin-4-yl)-2-oxoquinoline-1(2*H*)-carboxylate (11.9 g, 27.9 mmol) was dissolved in dichloromethane (200 mL) and trifluoroacetic acid (TFA) (30 mL) was added slowly, which led to release of gas and increase in temperature. The mixture was stirred at room temperature for 1 h and poured onto a vigorously stirred mixture of 2 M sodium hydroxide and ice and stirred for 5 min. A very fine precipitate was formed that was filtered off, leaving an off-white residue on the filter. This residue was dissolved in THF (1 L) and ethyl acetate (0.75 L) and dried with magnesium sulphate, filtered and concentrated in vacuo. The residue was dissolved in methanol (100 mL) and concentrated in vacuo to yield 7-(1,2,3,6-tetrahydropyridin-4-yl)quinolin-2(1*H*)-one as a foamy solid (8.23 g, 91%) that contained residual solvent as observed by ^{1}H NMR (methanol and 2,6-di-tert-butyl-4-methylphenol used as additive in the THF). The crude product was used in the next step without further purification. ^{1}H NMR (500 MHz, DMSO-d_6) δ 11.68 (brs, 1H), 7.87 (d, 1H), 7.60 (d, 1H), 7.31 (s, 1H), 7.29 (d, 1H), 6.45 (d, 1H), 6.32 (s, 1H), 3.47-3.44 (m, 2H), 2.99 (t, 2H), 2.41-2.36 (m, 2H), one NH was not resolved.

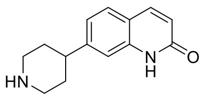

7-(Piperidin-4-yl)quinolin-2(1H)-one. 7-(1,2,3,6-Tetrahydropyridin-4-yl)quinolin-2(1*H*)-one (8.23 g, 25.5 mmol) was dissolved in a mixture of ethanol (350 mL) and THF (75 mL). Platinum(IV) oxide (0.72 g, 3.18 mmol) was added to the mixture that was hydrogenated at 1.05 bar hydrogen on a Parr apparatus at room temperature for 2.5 day. The mixture was filtered, and the solvent was concentrated in vacuo. The residual oil was purified by column flash chromatography (eluent: ethanol/triethylamine 19:1) to afford 7-(piperidin-4-yl)quinolin-2(1*H*)-one as a white powder after concentration in vacuo and used in the next step without further purification. ^{1}H NMR (500 MHz, DMSO-d_6) δ 11.70 (brs, 1H), 7.85 (d, 1H), 7.58 (d, 1H), 7.14 (s, 1H), 7.06 (d, 1H), 6.43 (d, 1H), 3.13 (t, 2H), 2.75-2.65 (m, 3H), 1.77 (d, 2H), 1.62-1.52 (m, 2H), one NH was not resolved.

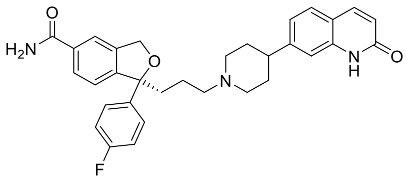

(*S*)-1-(4-Fluorophenyl)-1-(3-(4-(2-oxo-1,2-dihydroquinolin-7-yl)piperidin-1-yl)propyl)-1,3-dihydroisobenzofuran-5-carboxamide (Lu AF60097). (*S*)-1-(3-Chloropropyl)-1-(4-fluorophenyl)-1,3-dihydroisobenzofuran-5-carboxamide (0.58 g, 1.74 mmol) was dissolved in DMF (10 mL) and added 7-(piperidin-4-yl)quinolin-2(1*H*)-one (0.40 g, 1.58 mmol). Potassium carbonate (0.33 g, 2.37 mmol) and sodium iodide (2.36 g, 15.77 mmol) was added, and the resulting mixture was heated at 90 °C for 3 h and stirred at room temperature for an additional 16 h. The mixture was poured onto brine and extracted with a mixture of ethyl acetate and THF (2:1). The combined organic phase was washed with brine, dried over magnesium sulphate and concentrated in vacuo. The residual oil was purified by column flash chromatography (eluent: ethyl acetate/ethanol/triethylamine 14:5:1) to afford crude title compound after concentration in vacuo. The crude compound was taken up in a mixture of THF (10 mL) and methanol

(1 mL) and cooled on an ice bath. A solution of 5% oxalic acid in THF was added dropwise to the compound solution until the resulting solution was slightly acidic (pH approximately 4). A white precipitate formed, which was collected by filtration giving a white powder (0.29 g, 30%). ^{1}H NMR (600 MHz, DMSO-d_6) δ 11.75 (brs, 1H), 7.98 (s, 1H), 7.86 (d, 1H), 7.83 (d, 1H), 7.79 (s, 1H), 7.63-7.58 (m, 4H), 7.40 (s, 1H), 7.20-7.15 (m, 2H), 7.12 (s, 1H), 7.05 (d, 1H), 6.44 (d, 1H), 5.22 (d, 1H), 5.16 (d, 1H), 3.29 (brs, 2H), 2.86 (brs, 2H), 2.80-2.63 (m, 3H), 2.22 (t, 2H), 1.92-1.75 (m, 4H), 1.60-1.40 (m, 2H). HRMS calcd for $C_{32}H_{32}FN_3O_3$+H: 526.2500; Found: 526.2502. LC/MS (method 550): 100%, RT = 0.49 min. Enantiomer ratio (method A): >99.5%, RT = 3.51 min.

The following compounds were prepared in a similar manner.

(*RS*)-1-(4-Fluorophenyl)-1-(3-(4-(2-oxo-1,2-dihydroquinolin-7-yl)piperidin-1-yl)propyl)-1,3-dihydroisobenzofuran-5-carboxamide. Prepared starting from (*RS*)-1-(3-chloropropyl)-1-(4-fluorophenyl)-1,3-dihydroisobenzofuran-5-carboxamide (0.42 g, 1.13 mmol) and 7-(piperidin-4-yl)quinolin-2(1*H*)-one (0.25 g, 0.99 mmol) to yield 0.14 g, 24%. ^{1}H NMR is not reported as like spectrum for Lu AF60097. HRMS calcd for $C_{32}H_{32}FN_3O_3$+H: 526.2500; Found: 526.2507. LC/MS (method 550): 98%, RT = 0.49 min.

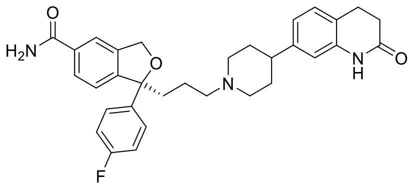

(*S*)-1-(4-Fluorophenyl)-1-(3-(4-(2-oxo-1,2,3,4-tetrahydroquinolin-7-yl)piperidin-1-yl)propyl)-1,3-dihydroisobenzofuran-5-carboxamide. Prepared starting from (*S*)-1-(3-chloropropyl)-1-(4-fluorophenyl)-1,3-dihydroisobenzofuran-5-carboxamide (0.44 g, 1.04 mmol) and 7-(piperidin-4-yl)-3,4-dihydroquinolin-2(1*H*)-one (ACS Scientific Inc.) (0.30 g, 1.30 mmol) to yield 0.40 g, 61%. ^{1}H NMR (600 MHz, DMSO-d_6) 10.10 (s, 1H), 7.98 (s, 1H), 7.83 (d, 1H), 7.79 (s, 1H), 7.64-7.58 (m, 3H), 7.40 (s, 1H), 7.20-7.15 (m, 2H), 7.10 (d, 1H), 6.74 (s, 1H), 6.66 (s, 1H), 5.23 (d, 1H), 5.16 (d, 1H), 3.43 (brs, 2H), 3.04 (brs, 2H), 2.98-2.85 (m, 2H), 2.81 (t, 2H), 2.72-2.63 (m, 1H), 2.42 (t, 2H), 2.22 (t, 2H), 1.91-1.70 (m, 4H), 1.65-1.44 (m, 2H). HRMS calcd for $C_{32}H_{34}FN_3O_3$+H: 528.2657; Found:

528.2660. LC/MS (method 450): 100%, RT = 0.47 min. Enantiomer ratio (method B): 99.3%, RT = 6.98 min.

(*R*)-1-(4-Fluorophenyl)-1-(3-(4-(2-oxo-1,2,3,4-tetrahydroquinolin-7-yl)piperidin-1-yl)propyl)-1,3-dihydroisobenzofuran-5-carboxamide. Prepared starting from (*R*)-1-(3-chloropropyl)-1-(4-fluorophenyl)-1,3-dihydroisobenzofuran-5-carboxamide (0.64 g, 1.53 mmol) and 7-(piperidin-4-yl)-3,4-dihydroquinolin-2(1*H*)-one (ACS Scientific Inc.) (0.44 g, 1.92 mmol) to yield 0.65 g, 69%. ^{1}H NMR is not reported as like spectrum for (*S*)-1-(4-fluorophenyl)-1-(3-(4-(2-oxo-1,2,3,4-tetrahydroquinolin-7-yl)piperidin-1-yl)propyl)-1,3-dihydroisobenzofuran-5-carboxamide. HRMS calcd for $C_{32}H_{34}FN_3O_3$+H: 528.2657; Found: 528.2661. LC/MS (method 450): 100%, RT = 0.46 min. Enantiomer ratio (method B): >99.5%, RT = 5.21 min.

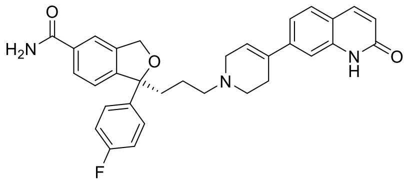

(*S*)-1-(4-Fluorophenyl)-1-(3-(4-(2-oxo-1,2-dihydroquinolin-7-yl)-3,6-dihydropyridin-1(2H)-yl)propyl)-1,3-dihydroisobenzofuran-5-carboxamide. Prepared starting from (*S*)-1-(3-chloropropyl)-1-(4-fluorophenyl)-1,3-dihydroisobenzofuran-5-carboxamide (0.80 g, 1.92 mmol) and 7-(1,2,3,6-tetrahydropyridin-4-yl)quinolin-2(1*H*)-one (0.55 g, 2.43 mmol) to yield 0.26 g, 22%. ^{1}H NMR (500 MHz, DMSO-d_6) 11.66 (s, 1H), 7.94 (s, 1H), 7.86 (d, 1H), 7.82 (d, 1H), 7.77 (s, 1H), 7.64-7.55 (m, 4H), 7.35 (s, 1H), 7.31-7.24 (m, 2H), 7.15 (t, 2H), 6.44 (d, 1H), 6.23 (s, 1H), 5.20 (d, 1H), 5.14 (d, 1H), 3.00 (brs, 2H), 2.57-2.50 (m, 2H), 2.43 (brs, 2H), 2.36 (t, 2H), 2.24-2.16 (m, 2H), 1.46-1.27 (m, 2H). HRMS calcd for $C_{32}H_{30}FN_3O_3$+H: 524.2344; Found: 524.2354. LC/MS (method 450): 100%, RT = 0.43 min. Enantiomer ratio is estimated to be >99% based on its synthesis from enantiomeric pure (*S*)-1-(3-chloropropyl)-1-(4-fluorophenyl)-1,3-dihydroisobenzofuran-5-carboxamide similarly to the synthesis of (*S*)-1-(4-fluorophenyl)-1-(3-(4-(2-oxo-1,2-dihydroquinolin-7-yl)piperidin-1-yl)propyl)-1,3-dihydroisobenzofuran-

5-carboxamide (Lu AF60097) and (*S*)-1-(4-fluorophenyl)-1-(3-(4-(2-oxo-1,2,3,4-tetrahydroquinolin-7-yl)piperidin-1-yl)propyl)-1,3-dihydroisobenzofuran-5-carboxamide.

(*S*)-[^{3}H]-1-(4-Fluorophenyl)-1-(3-(4-(2-oxo-1,2-dihydroquinolin-7-yl)piperidin-1-yl)propyl)-1,3-dihydroisobenzofuran-5-carboxamide ([^{3}H]Lu AF60097). Prepared at Red Glead Discovery AB, Lund, Sweden by treating (*S*)-1-(4-fluorophenyl)-1-(3-(4-(2-oxo-1,2-dihydroquinolin-7-yl)-3,6-dihydropyridin-1(2H)-yl)propyl)-1,3-dihydroisobenzofuran-5-carboxamide with 500 mbar tritium gas (generated from uranium tritide) and 10% palladium on carbon for 2 h in a mixture of ethanol and acetic acid. The crude product was purified by high performance (HP)LC (column: XTerra C18 (4.6x100 mm, 5 µM); gradient: 5-95 % B in 8 min; buffer A: water + 0.1% diethylamine and buffer B: acetonitrile + 0.1% diethylamine) to give a batch of 0.8 mCi [^{3}H]Lu AF60097 in 0.1 mL ethanol. The specific activity was determined to be 28 Ci/mmol.
