# Supplementary material for: The mechanism of a high-affinity allosteric inhibitor of the serotonin transporter
Source: Nat Commun. 2020 Mar 20;11:1491. doi: 10.1038/s41467-020-15292-y (PMC7083837; doi:10.1038/s41467-020-15292-y)
Supplement: Supplementary file 5 — Reporting Summary [file 41467_2020_15292_MOESM5_ESM.pdf]

nature research

Corresponding author(s): B. Bang-Andersen, L. Shi, C.J. Loland

Last updated by author(s): Feb 18, 2020

Reporting Summary

Nature Research wishes to improve the reproducibility of the work that we publish. This form provides structure for consistency and transparency in reporting. For further information on Nature Research policies, see Authors & Referees and the Editorial Policy Checklist.

Statistics

For all statistical analyses, confirm that the following items are present in the figure legend, table legend, main text, or Methods section.

n/a	Confirmed	
☐	☒	The exact sample size (*n*) for each experimental group/condition, given as a discrete number and unit of measurement
☐	☒	A statement on whether measurements were taken from distinct samples or whether the same sample was measured repeatedly
☐	☒	The statistical test(s) used AND whether they are one- or two-sided<br>*Only common tests should be described solely by name; describe more complex techniques in the Methods section.*
☐	☒	A description of all covariates tested
☐	☒	A description of any assumptions or corrections, such as tests of normality and adjustment for multiple comparisons
☐	☒	A full description of the statistical parameters including central tendency (e.g. means) or other basic estimates (e.g. regression coefficient) AND variation (e.g. standard deviation) or associated estimates of uncertainty (e.g. confidence intervals)
☐	☒	For null hypothesis testing, the test statistic (e.g. *F*, *t*, *r*) with confidence intervals, effect sizes, degrees of freedom and *P* value noted<br>*Give P values as exact values whenever suitable.*
☒	☐	For Bayesian analysis, information on the choice of priors and Markov chain Monte Carlo settings
☒	☐	For hierarchical and complex designs, identification of the appropriate level for tests and full reporting of outcomes
☒	☐	Estimates of effect sizes (e.g. Cohen's *d*, Pearson's *r*), indicating how they were calculated

Our web collection on statistics for biologists contains articles on many of the points above.

Software and code

Policy information about availability of computer code

Data collection	Pharmacological experiments: Prism 8<br>Molecular dynamics simulations: Desmond MD systems (D. E. Shaw Research, New York, NY) with OPLS3 force field; Molecular docking and MM/GBSA calculations: Schrodinger suite.
Data analysis	Prism 8 and MDTraj

For manuscripts utilizing custom algorithms or software that are central to the research but not yet described in published literature, software must be made available to editors/reviewers. We strongly encourage code deposition in a community repository (e.g. GitHub). See the Nature Research guidelines for submitting code & software for further information.

Data

Policy information about availability of data

All manuscripts must include a data availability statement. This statement should provide the following information, where applicable:

- Accession codes, unique identifiers, or web links for publicly available datasets
- A list of figures that have associated raw data
- A description of any restrictions on data availability

All relevant data are available from the authors. The source data underlying Figs 1b-e, 3b+c, 4b, 5a, 6 and Supplementary fig 6 are provided as a Source Data File.

Field-specific reporting

Please select the one below that is the best fit for your research. If you are not sure, read the appropriate sections before making your selection.

☒ Life sciences ☐ Behavioural & social sciences ☐ Ecological, evolutionary & environmental sciences

For a reference copy of the document with all sections, see nature.com/documents/nr-reporting-summary-flat.pdf

Life sciences study design

All studies must disclose on these points even when the disclosure is negative.

Sample size	For the biological samples, we are not able to determine the statistical power of the experiment beforehand. We aim for sample sizes of 3, and 5 for central data. For behavioral data acquisition, we add sample sizes until the power is sufficiently high.
Data exclusions	Data exclusion was determined with Prism 8.
Replication	All attempts of replication were successful. Otherwise it would not have been published.
Randomization	We perform experiments in random order so the sequence of outcome is different between experiments.
Blinding	When working with molecular dynamics simulations, molecular biology and cell lines, the experiments can not be blinded.

Reporting for specific materials, systems and methods

We require information from authors about some types of materials, experimental systems and methods used in many studies. Here, indicate whether each material, system or method listed is relevant to your study. If you are not sure if a list item applies to your research, read the appropriate section before selecting a response.

Materials & experimental systems

n/a	Involved in the study	
☒	☐	Antibodies
☐	☒	Eukaryotic cell lines
☒	☐	Palaeontology
☐	☒	Animals and other organisms
☒	☐	Human research participants
☒	☐	Clinical data

Methods

n/a	Involved in the study	
☒	☐	ChIP-seq
☒	☐	Flow cytometry
☒	☐	MRI-based neuroimaging

Eukaryotic cell lines

Policy information about cell lines

Cell line source(s)	The cells were a generous gift from Prof. U. Gether, Dept of Neuroscience, University of Copenhagen. He recieved them 20 years ago form Prof. T Schwartz, U. Copenhagen.
Authentication	None of the cells were used authenticated
Mycoplasma contamination	All the cells were tested negative for mycoplasma infection
Commonly misidentified lines (See ICLAC register)	none

Animals and other organisms

Policy information about studies involving animals; ARRIVE guidelines recommended for reporting animal research

Laboratory animals	male Sprague Dawley rats
Wild animals	no wild animals were used
Field-collected samples	n.a.
Ethics oversight	All animal experiments were approved by the Danish Animal Experiments Inspectorate

Note that full information on the approval of the study protocol must also be provided in the manuscript.
