# Supplementary material for: The mechanism of a high-affinity allosteric inhibitor of the serotonin transporter
Source: Nat Commun. 2020 Mar 20;11:1491. doi: 10.1038/s41467-020-15292-y (PMC7083837; doi:10.1038/s41467-020-15292-y)
Supplement: Supplementary file 6 — Description of Additional Supplementary Files [file 41467_2020_15292_MOESM6_ESM.pdf]

Title: Supplementary Movie 1. The quinolinone moiety of S2:Lu AF60097 can protrude into a sub-pocket near the tip of the EL4b in the presence of S1:IMI.
Description: Representative trajectory of "S1:IMI/S2:Lu AF60097" (3.6 μs) simulation show varied dynamics of the quinolinone moiety of S2:Lu AF60097. In the presence of S1:IMI, this moiety protrudes into a sub-pocket near the tip of the EL4b. This is not possible in the presence of S1:S-CIT (Supplementary Movie 2). The S1 and S2 ligands are in sphere representations and in the same color scheme as in Fig. 3c,d.

Title: Supplementary Movie 2. The quinolinone moiety of S2:Lu AF60097 can not protrude into a sub-pocket near the tip of the EL4b in the presence of S1:S-CIT.
Description: Representative trajectories of "S1:S-CIT/S2:Lu AF60097" (3.0 μs) simulation show varied dynamics of the quinolinone moiety of S2:Lu AF60097. In the presence of S1:S-CIT, this moiety cannot protrude into a sub-pocket near the tip of the EL4b as is allowed in the S1-IMI condition (Supplementary Movie 1). The S1 and S2 ligands are in sphere representations and in the same color scheme as Fig. 3c,d.
